# Gut Microbes Reports – highlighting the value in team and translational science

**DOI:** 10.1080/29933935.2023.2270377

**Published:** 2023-12-15

**Authors:** Christopher Staley

The field of microbiome research began about 25 years ago with the emergence of next-generation metagenomics sequencing technologies.^1^ The field advanced exponentially thereafter with multiple active consortia characterizing almost every environment and body site and raising calls for more rigorous experimental design and data collection standards by 2012.^2^ By 2018, teams led by Rob Knight^3^ and Jack Gilbert^4^ had reviewed best practices and current understanding of at least the intestinal microbiome in human health, marking the end of the infancy and the beginning of the adolescence of the field. However, as better frameworks^5^ are proposed and challenges for standards in data collection are addressed,^6^ we are beginning to realize that early enthusiasm may have resulted in inadvertent overinterpretation of microbiome findings.^7^ Further, advances in sequencing technologies have revealed that organs previously thought to be sterile may harbor a microbiome,^8^ including the controversial possibility of a circulating microbiome.^9^ Thus, there remain many challenges and opportunities as we enter the troubled teen years of microbiome research.

A major challenge falls to new early investigators, post-doctoral associates, and graduate students who are trying to break into microbiome research or incorporate it as a secondary aim in interventional studies. Prominent microbiome-focused journals are increasingly interested in mechanism as way to filter high-quality studies, from among the thousands of submissions received, that are most likely to lead to translational advances. What is to be done with the hundreds of other studies that were rigorously performed but had small sample sizes, observational or correlative outcomes, or that generated more hypotheses than answers on their way to a translational breakthrough? Thus, we are launching *Gut Microbes Reports* as a sister journal to *Gut Microbes*. We hope to provide a home for rigorous studies related to the gut microbes that may not achieve the mechanistic level needed for “prime time”, but nonetheless report findings of scientific value. It is my hope that we can help to group smaller studies by field or theme, especially from multiple geographic areas, to begin building a global weight-of-evidence that will be more valuable to the global research community than a single-center (*n* = 30) study.

The challenge of translating microbiome science is another hurdle investigators are facing, literally. As a reviewer, editor, and, most frequently, graduate student committee member, I am reminded that learning both the computational techniques and how to describe the results are “experiential processes”, as one student called it, with multiple hiccups and clarifications along the way. Journals, though, also face the challenge of finding quality peer reviewers with concurrent expertise in microbiome and the disease, condition, or topic of interest, leading to long review times, inconsistent reviews, and, in some cases, editorial rejection due to a lack of expertise among the editorial team. To address these challenges, I agreed to take on the role of Editor-in-Chief of this new journal. We have recruited a talented editorial board, comprised of investigators with expertise across the microbiome spectrum, many of whom are junior faculty with hands-on experience working with microbiome data. We hope to guide a more empathetic review process where constructive reviews are preferred over those favoring a quick *reject*, in order to provide more equitable access to less experienced investigators and collaborative teams.

We hope that *Gut Microbes Reports* will provide a platform to highlight team and translational science that includes the gut microbes and help to provide a context to move even incremental findings from bench-to-bedside. We support research and review (narrative and systematic) articles, as well as case reports, rapid communications, methods, data notes, registered reports, and commentaries. We welcome topics ranging from descriptive and observational characterization of gut microbes to translational topics related to the microbiome in surgery, aging, neurodegenerative diseases, cancers, and nutrition (among others). Descriptions of new methods and computational tools with clear translational applications are also welcome, as are negative findings from studies that were otherwise well designed. We hope that you will help us support this new effort to expand and unite our global community of microbiome researchers and further untangle the roles of the gut microbes in health and disease. Submission is now open.
